# Dietary Intervention with Resistant Starch-Rich Unripe Plantain Flour Restores Gut Microbiome–Metabolome Axis and Ameliorates Type 2 Diabetes in Rats

**DOI:** 10.3390/foods14233996

**Published:** 2025-11-21

**Authors:** Jinfeng Fu, Cancan Liu, Shiyun Tu, Hongjie Liu, Zixin Liu, Weidi He, Lu Dong, Ganjun Yi, Yiji Xia, Juan Wang, Ou Sheng

**Affiliations:** 1 Institute of Fruit Tree Research, Guangdong Academy of Agricultural Sciences, Key Laboratory of South Subtropical Fruit Biology and Genetic Resource Utilization, Ministry of Agriculture and Rural Affairs, Guangdong Provincial Key Laboratory of Science and Technology Research on Fruit Tree, Guangzhou 510640, China; 202310186432@mail.scut.edu.cn (J.F.); liucancan2919@163.com (C.L.); sy.tu.scut@outlook.com (S.T.); 18753403832@163.com (H.L.); zephyrine_liu@163.com (Z.L.); heweidi89@163.com (W.H.); yiganjun@vip.163.com (G.Y.); 2 School of Food Science and Engineering, South China University of Technology, Guangzhou 510641, China; dl-loulou@outlook.com; 3 Department of Biology, Hong Kong Baptist University, Hong Kong 999077, China; yxia@hkbu.edu.hk

**Keywords:** unripe plantain flour, gut microbiota, diabetes mellitus, short-chain fatty acids, bile acids, type 2 diabetes

## Abstract

Plantain (*Musa* spp., AAB group) possesses a complex triploid genetics originating from interspecific hybridization, which underlies its agronomic traits and nutritional composition, making it a vital global staple food crop. Unripe plantain flour (UPF), a rich source of resistant starch (RS), has demonstrated anti-diabetic properties in diabetic rats, yet its mechanisms of action remain unclear. This study investigated whether unripe plantain flour attenuates type 2 diabetic traits in rats made diabetic with a high-fat diet plus streptozotocin through regulation of the gut microbiome–metabolome axis, including short-chain fatty acids and bile acids. We found that UPF intervention significantly ameliorated gut microbiota dysbiosis. It enriched beneficial bacteria, particularly SCFA producers (*Lachnoclostridium*, *Blautia*, *Butyricicoccus*) and others (*Bifidobacterium*, *Akkermansia*), while inhibiting harmful genera (*Romboutsia*, *Allobaculum*). Consequently, UPF altered bile-acid composition by lowering hydrophobic species (e.g., cholic acid and deoxycholic acid) while elevating hydrophilic species (e.g., ursodeoxycholic acid and tauroursodeoxycholic acid). It also enhanced the excretion of secondary bile acids (lithocholic acid). These coordinated changes in the gut ecosystem are conducive to improved glycolipid metabolism. Spearman correlation analysis further reinforced the close relationships between the altered microbiota and metabolites. Our results elucidate that UPF exerts its anti-diabetic effects by remodeling the gut microbiota and modulating its associated metabolites, highlighting a novel dietary intervention strategy for diabetes management.

## 1. Introduction

Diabetes mellitus comprises a group of chronic metabolic diseases marked by persistent hyperglycemia arising from impaired insulin secretion, impaired insulin action, or both [1]. The global prevalence of diabetes continues to rise alarmingly and is associated with severe macro- and microvascular complications, contributing to significant morbidity and mortality worldwide [2]. Current management strategies, including pharmacological interventions, dietary modifications, and lifestyle adjustments, often remain insufficient due to limitations in efficacy, accessibility, and sustainability [3]. Therefore, there is growing interest in exploring natural dietary interventions that target underlying metabolic pathways, particularly those involving the gut microbiota and its metabolites, as novel approaches for diabetes management.

Accumulating evidence indicates that diabetes is closely associated with compositional and functional alterations in the gut microbiota—a condition referred to as gut dysbiosis [4,5]. For instance, studies have reported microbial shifts such as an increased abundance of *Proteobacteria* and a decreased ratio of *Firmicutes* to *Bacteroidetes* in streptozotocin (STZ)-induced diabetes [6,7]. Specific beneficial bacteria, including acetate-producing Blautia, have been found to be reduced in diabetic fatty rats [1]. These microbial changes in gut bacteria influence the generation of SCFAs and bile acids, and these metabolites are fundamental mediators of glucose homeostasis, insulin sensitivity, and host inflammation [8,9]. Mechanisms include promotion of glycogen synthesis, inhibition of gluconeogenesis, reduction of lipid accumulation, and preservation of β-cell function [10]. Thus, targeting the gut microbiota-metabolite axis represents a promising therapeutic strategy for diabetes.

Plantain (*Musa* spp., AAB group), a distinct triploid hybrid derived from *Musa acuminata* (AA) and *Musa balbisiana* (BB), is among the world’s most important staple food crops [11]. Its significance is particularly pronounced across Africa and Central and South America, where it serves as an essential dietary staple and a key source of carbohydrates for millions of people [12]. Unlike dessert bananas, plantains are particularly rich in starch, especially when unripe. Unripe plantain flour (UPF) contains high levels of resistant starch (RS), a type of indigestible carbohydrate that resists hydrolysis in the small intestine and undergoes fermentation in the colon [13,14]. This process modulates the gut microbiota and enhances the production of beneficial metabolites, including SCFAs, which have been implicated in improved metabolic health [15]. Previous studies have shown that RS supplementation can enrich beneficial bacteria such as *Ruminococcus bromii* and *Bifidobacterium adolescentis*, alter bile acid metabolism, and increase SCFA production [15,16]. These findings suggest that RS may ameliorate diabetes symptoms through microbial-mediated mechanisms.

Although previous studies, including our own, have demonstrated the hypoglycemic effects of UPF in diabetic models [17,18,19]—such as improved body weight, food intake, serum biochemical profiles, and insulin resistance (shown in Table S1)—the specific mechanisms whereby UPF modulates gut microbiota and associated metabolites remain inadequately elucidated. In particular, simultaneous investigations of microbial shifts and metabolic responses following UPF intervention are lacking.

Therefore, the present study aimed to know how UPF affects the gut microbiome–metabolome axis in a diabetic rat model induced by a high-fat diet combined with streptozotocin. We profiled community composition, quantified short-chain fatty acids and bile acids, and examined links between bacterial taxa and metabolic indicators. Our results clarify mechanisms underlying the antidiabetic actions of UPF and support its potential as a microbiota-directed functional food for diabetes prevention and management.

## 2. Materials and Methods

### 2.1. Preparation and Characterization of UPF

**Figure 1:**
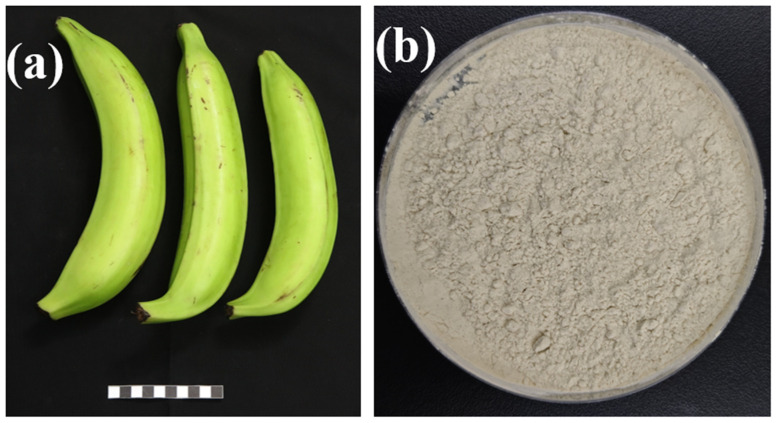
The images of Plantain (*Musa* spp., AAB group) fruit (**a**) and the unripe plantain flour (UPF) (**b**). (Note: Each black and white square in Figure 1a represents 1 cm).

Green mature fruits of plantain (*Musa* spp., AAB group) were obtained from the Institute of Fruit Tree Research, Guangdong Academy of Agricultural Sciences (Guangzhou, China). Uniform fruits were selected, washed, and manually peeled. The pulp was sliced, dried in a hot-air oven at 60 °C, and then milled into a fine powder. The images of Plantain (*Musa* spp., AAB group) and unripe plantain flour (UPF) are shown in Figure 1. The powder was passed through a 100-mesh sieve to obtain homogeneous UPF. The nutritional composition of UPF was characterized following our previously published protocol [20]. The nutritional composition of the resulting UPF, particularly its high RS content, was analyzed and is presented in Table S2.

### 2.2. Animals and Experimental Design

A total of 120 male Sprague–Dawley rats (specific pathogen-free; initial body weight 90–120 g) were obtained from the Guangdong Medical Laboratory Animal Center. The normal chow diet and the high-sugar, high-fat diet were supplied by the Experimental Animal Center of South China Agricultural University and the Guangdong Medical Laboratory Animal Center, respectively. On a weight basis, the normal chow contained nitrogen-free extract 55%, crude protein 18%, moisture 10%, crude ash 8%, crude fiber 5%, crude fat 4%, calcium 1–2%, and phosphorus 0.6–1.2%. The high-sugar, high-fat diet was compounded as follows (*w*/*w*): 47.6% normal chow, 10% lard, 20% sucrose, 15% egg yolk powder, 5% casein, 1.2% cholesterol, 0.2% sodium cholate, 0.6% calcium bicarbonate, and 0.4% stone powder.

### 2.3. Induction of Diabetes and Group Assignment

Following the China Food and Drug Administration guidelines, diabetes was produced by feeding a high-sugar, high-fat diet together with repeated low-dose streptozotocin injections. A cohort of 120 male Sprague–Dawley rats was maintained under standardized conditions (21 ± 2 °C; 60–70% humidity; 12 h/12 h light–dark) at the Experimental Animal Center of South China Agricultural University, four animals per cage with ad libitum food and water [21]. After a 7-day acclimatization period, rats were randomized to either a normal chow group (N = 24) or a high-sugar, high-fat diet group (N = 96).

After 6 weeks on the HSHF diet, the rats were fasted for 12 h (with free access to water) and then received intraperitoneal injections of STZ to induce diabetes. The diabetic model was established with minor modifications based on previously described methods [22,23,24,25]. Briefly, STZ was dissolved in ice-cold citrate buffer (0.1 mmol/L, pH 4.4) at a concentration of 10 mg/mL and administered intraperitoneally at a dose of 30 mg/kg body weight. Rats assigned to the diabetes model received an intraperitoneal injection of STZ dissolved in freshly prepared citrate buffer. Fasting blood glucose (FBG) was measured 72 h (3 days) after the first injection. Animals that did not meet the pre-specified hyperglycemia criterion then received a second intraperitoneal STZ injection, and FBG was re-assessed 72 h later. Animals failing to meet the criterion after two injections were excluded from the study. Healthy (control) rats received a single intraperitoneal injection of citrate buffer at the same time point as the first STZ injection in the model groups, and FBG was measured 72 h thereafter. All rats underwent tail-vein blood collection 72 h after injection, and animals were fasted for 12 h before sampling, with water provided ad libitum. FBG levels were measured using a Roche ACCU-CHEK Active glucometer. Rats with FBG levels ≥ 11.1 mmol/L were considered diabetic and included in the subsequent experiment. A total of 48 rats successfully met the diabetic criteria.

**Figure 2:**
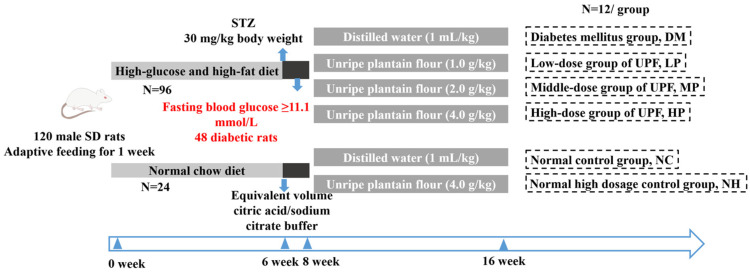
Schematic illustration of animal experimental protocol and design. (Note: Black indicates the model induction period; Gray indicates the diet and unripe plantain flour (UPF) intervention period; Black dashed boxes denote the group labels).

The eligible diabetic rats (N = 48) were randomly distributed into four treatment groups, and the normoglycemic rats (N = 24) were allocated into two control groups, yielding a total of six experimental groups (with 12 rats per group) as follows: the Normal Control (NC) group consisted of healthy rats maintained on a standard chow diet and administered distilled water (1 mL/kg) by oral gavage; the Normal High-dose (NH) group comprised healthy rats fed the standard diet and supplemented with a high dose of UPF at 4.0 g/kg; the Diabetic Model (DM) group included diabetic rats fed a HSHF diet and given distilled water (1 mL/kg); the Low-dose UPF (LP) group contained diabetic rats maintained on the HSHF diet and treated with UPF at 1.0 g/kg; the Middle-dose UPF (MP) group involved diabetic rats fed the HSHF diet and administered UPF at 2.0 g/kg; and the High-dose UPF (HP) group consisted of diabetic rats fed the HSHF diet and supplemented with UPF at 4.0 g/kg. NC and DM groups received distilled water by oral gavage at 1 mL/kg as vehicle to match the dosing procedure used in UPF groups. All animals had ad libitum access to standard drinking water throughout the experiment. The intervention lasted for 8 weeks. A schematic overview of the experimental design is presented in Figure 2.

Daily food intake, water consumption, and urine output were monitored at fixed time points throughout the intervention period, and body weight was recorded once per week on a designated measurement day. For metabolic indicators, serum glucose concentration was determined using an automated biochemical analyzer (BS-380, Shenzhen Mindray Bio-Medical Electronics Co., Ltd., Shenzhen, China). Circulating insulin levels were quantified using commercially available ELISA kits (Wuhan Bioswamp Bio-Technology Co., Ltd., Wuhan, China). These measurements were used to evaluate glycemic control and insulin regulation in the experimental groups.

### 2.4. Sample Collection

Fresh fecal samples were collected from all rats on a weekly basis and immediately stored at −80 °C for subsequent analysis. Following the 8-week intervention period, all rats were fasted for 12 h with free access to water. After final body weight measurement, the rats were euthanized under anesthesia. The intestinal tracts were rapidly dissected, placed in sterile cryogenic bags, quickly frozen in liquid nitrogen, and transferred to a −80 °C freezer for long-term preservation.

### 2.5. DNA Extraction and 16S rRNA Sequencing

Total genomic DNA was extracted from intestinal content samples (N = 5 per group) using the cetyltrimethylammonium bromide method [26]. DNA purity and concentration were assessed by 1% agarose gel electrophoresis. The bacterial 16S rRNA gene’s V3–V4 hypervariable region was targeted for amplification through PCR with the universal primers 341F (5′-CCTAYGGGRBGCASCAG-3′) and 806R (5′-GGACTACNNGGGTATCTAAT-3′). PCR products were verified by 2% agarose gel electrophoresis, pooled in equimolar ratios, and purified using the GeneJET Gel Recovery Kit (Thermo Fisher Scientific, Waltham, MA, USA). Amplicons of 400–450 bp were selected for library construction with the NEB Next^®^ Ultra™ DNA Library Prep Kit for Illumina (New England Biolabs, Ipswich, MA, USA). Qualified libraries were quantified using Qubit and sequenced on the Illumina HiSeq platform by Beijing Nuohe Zhiyuan Technology Co., Ltd., Beijing, China.

### 2.6. Determination of SCFAs

Fecal short-chain fatty acids were quantified using gas chromatography based on a published protocol with minor adjustments [27]. About 1 g of thawed feces was homogenized in 1 mL distilled water, vortexed for 2 min, and centrifuged at 12,000× *g* for 20 min at 4 °C. The supernatant was passed through a 0.22 µm aqueous-phase filter and injected into an Agilent 8860 GC (Agilent Technologies, Santa Clara, CA, USA) equipped with a flame ionization detector and an AE-FFAP capillary column (30 m × 0.25 mm × 0.25 µm; ATEO, Shanghai, China). Nitrogen served as the carrier gas at 1 mL/min. Injector and detector temperatures were 240 °C and 250 °C, respectively. The oven program was 110 °C (1 min), ramp to 170 °C at 8 °C/min, then to 220 °C at 10 °C/min.

### 2.7. Quantification of Bile Acids

Fecal bile acid extraction and analysis were performed according to a reported protocol with minor adjustments [28]. About 1 g of fecal material was homogenized in 2 mL methanol, vortexed thoroughly, and centrifuged at 6000× *g* for 15 min at 4 °C. The supernatant was passed through a 0.22 µm membrane filter and analyzed by UPLC–MS/MS (Bruker, Berlin, Germany). Data processing and analyses were performed using the IsotopePattern and DataAnalysis in the Bruker Daltonics 3.1 software package.

UPLC-MS/MS analysis was performed with minor modifications [28,29]. Chromatographic separation was performed on an Agilent SB-C18 RRHD column (2.1 × 50 mm, 1.8 μm) maintained at 40 °C. The mobile phase comprised two components: (A) water containing 0.01% formic acid and (B) acetonitrile. A gradient elution program was employed with the following profile: 0–4 min (25% B), 4–9 min (25–30% B), 9–14 min (30–36% B), 14–18 min (36–38% B), 18–24 min (38–50% B), 24–32 min (50–75% B), 32–35 min (75–100% B), and 35–38 min (100–25% B). The mobile-phase flow rate was 0.25 mL/min, and the autosampler injection volume was 5 µL. Mass detection was conducted in negative electrospray ionization mode under the following conditions: ion source temperature 180 °C, voltage 3500 V, dry gas flow 4 L/min, and scan range 50–1000 *m*/*z*. Quantification was performed using external standard curves.

### 2.8. Bioinformatics Analysis

Raw 16S rRNA sequencing data were processed on the NovoMagic cloud platform. Operational taxonomic units (OTUs) were clustered at 97% similarity using Uparse v7.0.1001. Taxonomic annotation was performed against the SILVA132 SSUrRNA database using the mothur algorithm. Alpha diversity indices and UniFrac distances were calculated with QIIME v1.9.1. Beta diversity was visualized via principal coordinates analysis (PCoA) using R v2.15.3. Linear discriminant analysis effect size (LEfSe) was applied to identify differentially abundant taxa with an LDA score threshold of 3. We applied Spearman’s correlation analysis in R to assess relationships between microbial taxa and metabolic parameters.

### 2.9. Statistical Analysis

Data are expressed as mean ± standard deviation. Statistical analyses were conducted in SPSS 24.0 (IBM Corp., New York, NY, USA). After testing for homogeneity of variance, one-way ANOVA was used, followed by LSD for equal variances or Dunnett’s T3 for unequal variances. Significance was set at *p* < 0.05. Graphs were generated by Origin 9.1.

## 3. Results

### 3.1. Effects of UPF on Gut Microbiota Diversity in Diabetic Rats

The influence of UPF on gut microbial diversity was evaluated using Alpha diversity indices. Alpha diversity describes the diversity within an individual sample. Good’s coverage, which estimates sequencing completeness and indicates whether the majority of taxa present in the sample have been captured. Chao1 and ACE, which are richness estimators that infer the total number of taxa (including rare/low-abundance taxa) in the community. The Shannon index, which considers both richness and evenness, reflects not only how many taxa are present but also how evenly they are distributed within the sample. These indices together characterize the internal complexity of the gut microbial community in each group [30]. As summarized in Table 1, compared to the NC group, the DM group exhibited significant reductions in the ACE index by 41.25%, the Chao1 index by 43.33%, and the Shannon index by 14.10% (*p* < 0.05), indicating a substantial decrease in microbial richness and diversity due to diabetes induction. Although not statistically significant, the LP group showed increases in both ACE and Chao1 indices compared to the DM group, with values approaching those of the NC group (*p* > 0.05), suggesting that UPF intervention may help restore microbial abundance in diabetic rats.

**Figure 3:**
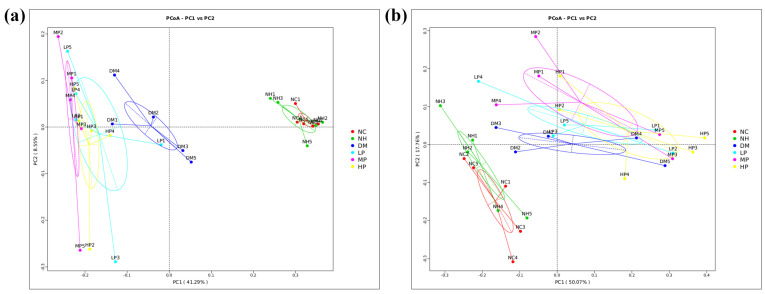
Principal coordinates analysis (PCoA) of gut microbiota β-diversity among groups. ((**a**): Unweighted UniFrac, (**b**): Weighted UniFrac).

Beta diversity was assessed using PCoA based on both Unweighted and Weighted UniFrac distances. Beta diversity measures differences in microbial community structure between samples or groups. Ordination and clustering based on beta diversity (e.g., PCoA, hierarchical clustering) indicate how similar or dissimilar the overall community composition is across treatment groups; samples that cluster closely share more similar microbiota profiles [31]. In the Unweighted PCoA (Figure 3a), which emphasizes presence-absence of taxa, principal coordinates 1 and 2 explained 41.29% and 8.55% of the total variance, respectively. Samples from the NC and NH groups clustered closely in the first quadrant, indicating high community similarity. In contrast, DM group samples were distributed across the second and fourth quadrants and were completely separated from NC samples, confirming structural alterations in the gut microbiota due to diabetes. The UPF intervention groups (LP, MP, and HP) were clearly distinguishable from the DM group, with separation distances following the order: MP > HP > LP, demonstrating that UPF administration partially restored the microbial community structure in a dose-dependent manner.

The Weighted UniFrac PCoA (Figure 3b), which considers phylogenetic abundance, showed higher explanatory values for PC1 (50.07%) and PC2 (17.76%). However, inter-group distances were reduced compared to the unweighted analysis. While the NC and DM groups remained separable, the distances between UPF intervention groups and the DM group were less pronounced, indicating that phylogenetic abundance influenced the perception of microbial structural differences.

### 3.2. Modulation of Gut Microbiota Composition by UPF

**Figure 4:**
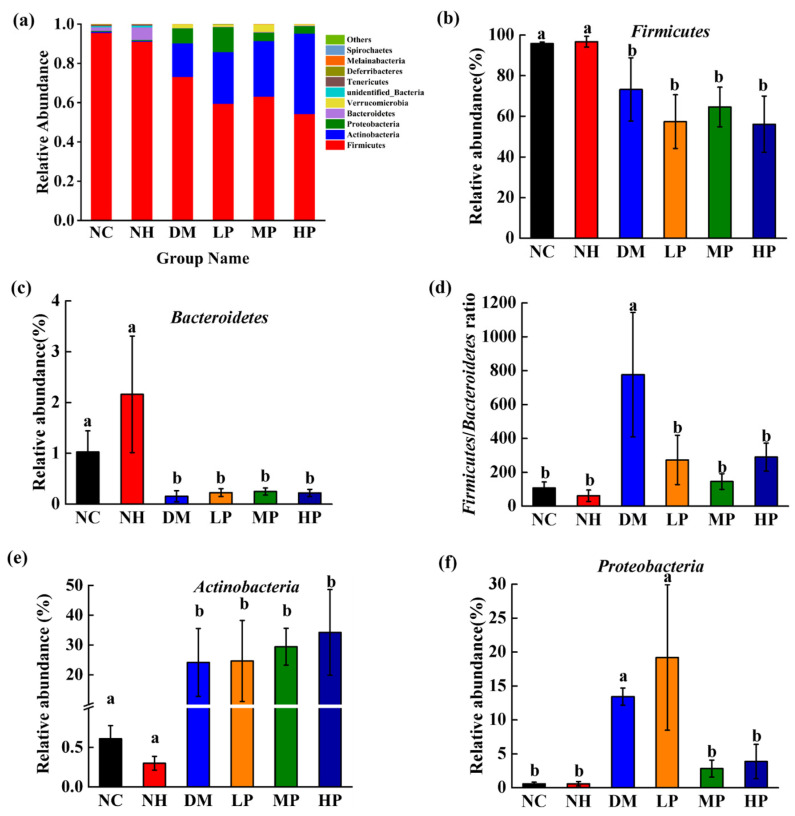
Effects of UPF on the structure of the gut microbiota of rats. (**a**) bacterial taxonomic profiling at the phylum level, (**b**) the relative abundance of Firmicutes, (**c**) the relative abundance of Bacteroidetes, (**d**) the ratio of Firmicutes to Bacteroidetes, (**e**) the relative abundance of Actinobacteria, (**f**) the relative abundance of Proteobacteria. Note: Different lowercase letters indicate significant differences among NC, NH, DM, LP, MP, and HP (one-way ANOVA; LSD or Dunnett’s T3 as appropriate; *p* < 0.05).

The relative abundances of major bacterial phyla are shown in Figure 4a. Quantitative analysis revealed that compared to the NC group, the DM group had significantly lower abundances of *Firmicutes* (Figure 4b) and *Bacteroidetes* (Figure 4c), resulting in a significantly higher *Firmicutes*/*Bacteroidetes* (F/B) ratio (Figure 4d; *p* < 0.05). Compared with the DM group, the F/B ratio was significantly lower in the UPF low-dose (LP), medium-dose (MP), and high-dose (HP) groups (*p* < 0.05), although individual phylum abundances were not significantly altered. Notably, UPF increased the relative abundance of *Bacteroidetes* in the NH group, but this effect was not observed in the DM groups. A likely explanation is that the effect of UPF is highly dependent on the host’s baseline gut microbiota and metabolic milieu [32]. In a healthy gut, UPF can promote the growth of *Bacteroidetes*. However, in diabetes, dysbiosis and metabolic dysfunction may impair the metabolism and utilization of resistant starch, thereby weakening the intervention effect.

The DM group also exhibited significantly increased abundances of *Actinobacteria* (Figure 4e) and *Proteobacteria* (Figure 4f) compared to NC (*p* < 0.05). While UPF intervention did not significantly affect *Actinobacteria* abundance, the MP and HP groups showed marked reductions in *Proteobacteria* abundance by 79.02% and 71.24%, respectively (*p* < 0.05), suggesting that *Proteobacteria* may serve as a potential diagnostic and therapeutic target for diabetes management. Additionally, at the phylum level, *Verrucomicrobia* showed an increase in the MP group compared with the DM group (Figure 4a). Genus-level inspection indicated that this change was primarily driven by *Akkermansia*.

**Figure 5:**
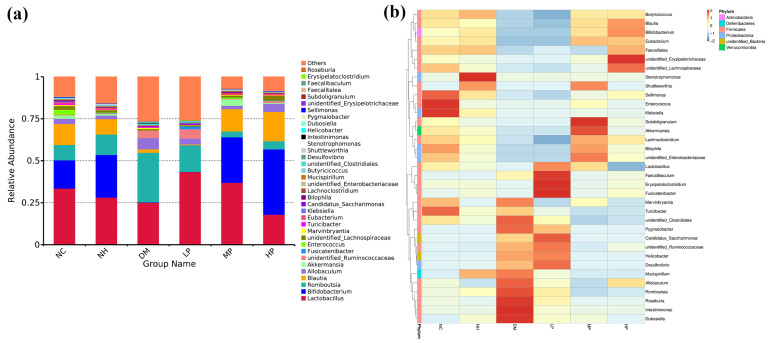
The bacterial taxonomic profiling (**a**) and the clustering heatmap of species abundance (**b**) at the genus level.

**Figure 6:**
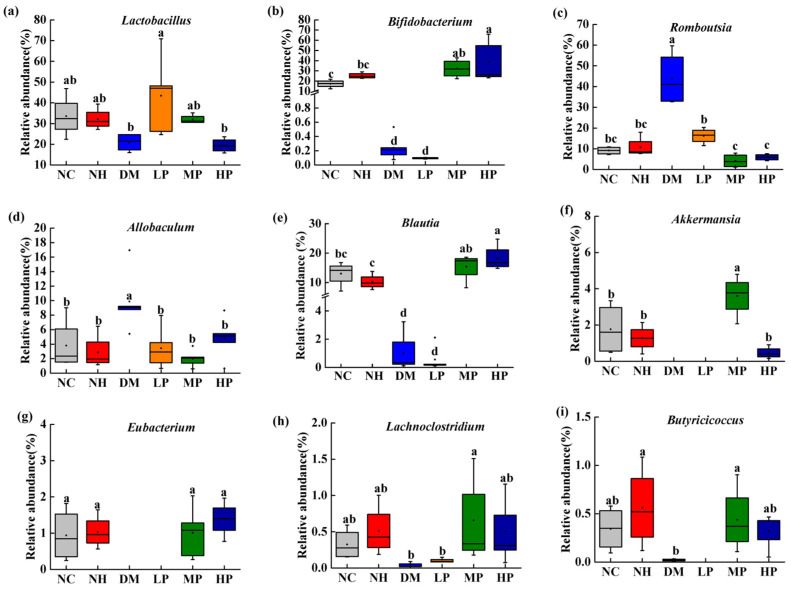
Effects of UPF on the relative abundance of different genera at the genus level. (**a**) *Lactobacillus*; (**b**) *Bifidobacterium*; (**c**) *Romboutsia*; (**d**) *Allobaculum*; (**e**) *Blautia*; (**f**) *Akkermansia*; (**g**) *Eubacterium*; (**h**) *Lachnoclostridium*; (**i**) *Butyricicoccus*. (Note: different lowercase letters indicate significant differences among NC, NH, DM, LP, MP, and HP (one-way ANOVA; LSD or Dunnett’s T3 as appropriate); *p* < 0.05).

Genus-level analysis revealed substantial alterations in microbial composition (Figure 5a). Heatmap analysis of the top 35 abundant species (Figure 5b) demonstrated that diabetes significantly altered the gut microbiota structure, which was partially restored by UPF intervention, particularly in the MP and HP groups. Detailed analysis of dominant genera (Figure 6) showed that compared to NC, the DM group had significantly reduced abundances of beneficial genera including *Bifidobacterium*, *Blautia*, *Akkermansia*, and *Eubacterium* (*p* < 0.05), while potentially harmful genera *Romboutsia* and *Allobaculum* were significantly increased (*p* < 0.05). UPF intervention significantly reversed these changes, increasing the abundances of *Lactobacillus*, *Bifidobacterium*, *Blautia*, *Akkermansia*, *Eubacterium*, *Lachnoclostridium*, and *Butyricicoccus* (*p* < 0.05), while reducing *Romboutsia* and *Allobaculum* (*p* < 0.05).

### 3.3. Identification of Differential Microbial Taxa

**Figure 7:**
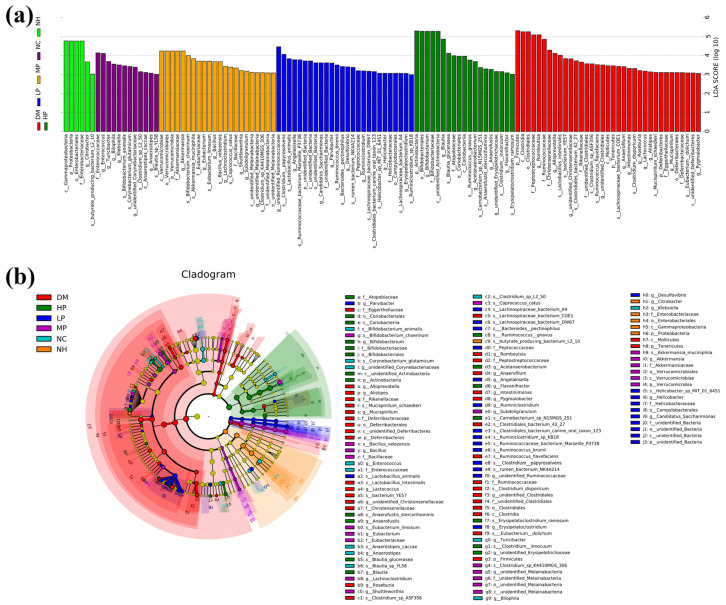
The gut microbiota phylotypes with the statistical difference in abundance among all the groups identified by linear discriminant analysis effect size (LEfSe) analysis. (**a**) LDA value distribution histogram (**b**) evolutionary branch diagram.

LEfSe analysis identified 120 differentially abundant bacterial taxa across all experimental groups at various taxonomic levels (Figure 7). The NC, NH, DM, LP, MP, and HP groups contained 12, 6, 35, 26, 22, and 19 unique taxa, respectively. At the genus level, the NC group was characterized by unique taxa including *Enterococcus*, *Turicibacter*, *Bilophila*, *Klebsiella*, *unidentified_Corynebacteriaceae*, and *Anaerostipes*. The NH group featured Citrobacter as its unique genus. The DM group showed 11 unique genera including *Romboutsia*, *Alloprevotella*, *unidentified_Christensenellaceae*, *unidentified_Clostridiales*, *Anaerofilum*, *Intestinimonas*, *Roseburia*, *Lactococcus*, *Alistipes*, *Mucispirillum*, and *Pygmaiobacter*. The LP group contained 8 unique genera: *unidentified_Ruminococcaceae*, *Angelakisella*, *Candidatus_Saccharimonas*, *Parvibacter*, *Desulfovibrio*, *Ruminiclostridium*, *Helicobacter*, and *Erysipelatoclostridium*. The MP group featured 7 unique genera including *Akkermansia*, *Eubacterium*, *Bacillus*, *Lachnoclostridium*, *Shuttleworthia*, *Subdoligranulum*, and *unidentified_Melainabacteria*. The HP group contained 5 unique genera: *Bifidobacterium*, *Blautia*, *Acetanaerobacterium*, *unidentified_Erysipelotrichacea*, and *Flavonifractor*. These taxa represent potential key mediators of UPF’s microbiota-modulating effects.

### 3.4. Effects of UPF on Fecal SCFAs

**Figure 8:**
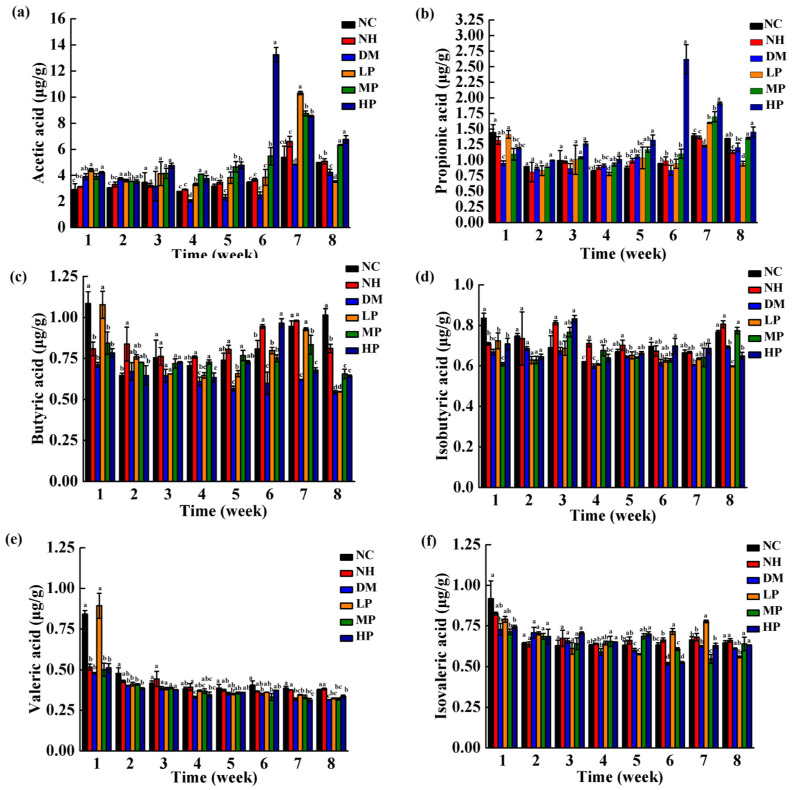
Effects of UPF on the concentrations of SCFAs in the feces of rats. (**a**) Acetic acid; (**b**) Propionic acid; (**c**) Butyric acid; (**d**) Isobutyric acid; (**e**) Valeric acid; (**f**) Isovaleric acid. (Note: different lowercase letters indicate significant differences among NC, NH, DM, LP, MP, and HP (one-way ANOVA; LSD or Dunnett’s T3 as appropriate); *p* < 0.05).

The changes in fecal SCFA concentrations across experimental groups are summarized in Figure 8. From weeks 2 to 8, in the NH group, the concentrations of acetic acid, propionic acid, valeric acid, and isovaleric acid showed no significant differences compared to the NC group (*p* > 0.05), except for propionic acid in the eighth week. In contrast, the concentrations of butyric acid (weeks 2–7) and isobutyric acid (weeks 3–8, except for week 6) were elevated in the NH group relative to NC. As butyric acid plays a critical role in host metabolic health—modulating lipid metabolism, glucose homeostasis, inflammation, and gut microbiota composition [33]—these results indicate that UPF supplementation enhances the production of specific SCFAs, particularly butyrate-related metabolites, even under non-diabetic conditions.

In the DM group, dynamic temporal changes in SCFA levels were observed. During the first week post-induction, all SCFAs except acetic acid decreased significantly compared to the NC group (*p* < 0.05). By weeks 2 and 3, however, most SCFA concentrations showed no significant difference from NC (*p* > 0.05), except valeric acid, which remained reduced in week 2. This transient recovery may reflect an acute compensatory response to diabetic stress. As diabetes progressed, sustained hyperglycemia and metabolic dysregulation led to significant reductions in SCFA concentrations from week 4 onward (*p* < 0.05). Butyric acid and valeric acid were particularly affected, showing persistently lower levels throughout weeks 4–8 (*p* < 0.05), except for valeric acid in the fifth week. These results suggest that diabetes selectively disrupts SCFA biosynthesis, with butyrate and valerate exhibiting the greatest susceptibility to metabolic impairment.

UPF intervention significantly counteracted diabetes-induced SCFA depletion. From weeks 4 to 8, all SCFAs except valeric acid showed marked increases in UPF-treated groups compared to the DM group (*p* < 0.05); valeric acid increased significantly only at week 8. The restorative effects of UPF became more pronounced in the later intervention phase (weeks 4–8) compared to the initial period (weeks 1–3), underscoring the time-dependent efficacy of dietary intervention in chronic diabetic states.

By the end of the 8-week intervention, notable dose-responsive improvements were observed in the MP and HP groups. Acetic acid concentrations increased by 49.34% and 59.43%, respectively (DM: 4.24 ± 0.23 µg/g; *p* < 0.05). Propionic acid increased by 12.33% and 20.49% (DM: 1.21 ± 0.07 µg/g; *p* < 0.05), and butyric acid rose by 20.59% and 18.18% (DM: 0.54 ± 0.01 µg/g; *p* < 0.05). Isobutyric acid increased by 11.67% in the MP group (DM: 0.69 ± 0.01 µg/g; *p* < 0.05), and valeric acid increased by 6.53% in the HP group (DM: 0.315 ± 0.001 µg/g; *p* < 0.05). In contrast, isovaleric acid levels remained unchanged across all UPF-treated groups (*p* > 0.05). These results demonstrate that UPF intervention—particularly at medium and high doses—effectively restores SCFA production in diabetic rats, with the most substantial effects observed for acetic, propionic, and butyric acids.

### 3.5. Modulation of Bile Acid Metabolism in the Feces of Rats

Bile acids, synthesized in the liver from cholesterol, support emulsification and uptake of dietary fats. They are secreted into bile, accumulate in the gallbladder, and are released into the duodenum during feeding. Approximately 95% are absorbed in the ileum and recirculated to the liver via enterohepatic transport. The remaining portion is metabolized by the gut microbiota in the colon to form secondary bile acids, which have antibacterial and cytotoxic properties and are ultimately excreted in feces [34]. Modulating bile acid metabolism—specifically enhancing hepatic conversion of cholesterol to bile acids, reducing intestinal reabsorption, and promoting fecal excretion of secondary bile acids—represents a promising strategy for managing cholesterol homeostasis in conditions of lipid excess.

**Figure 9:**
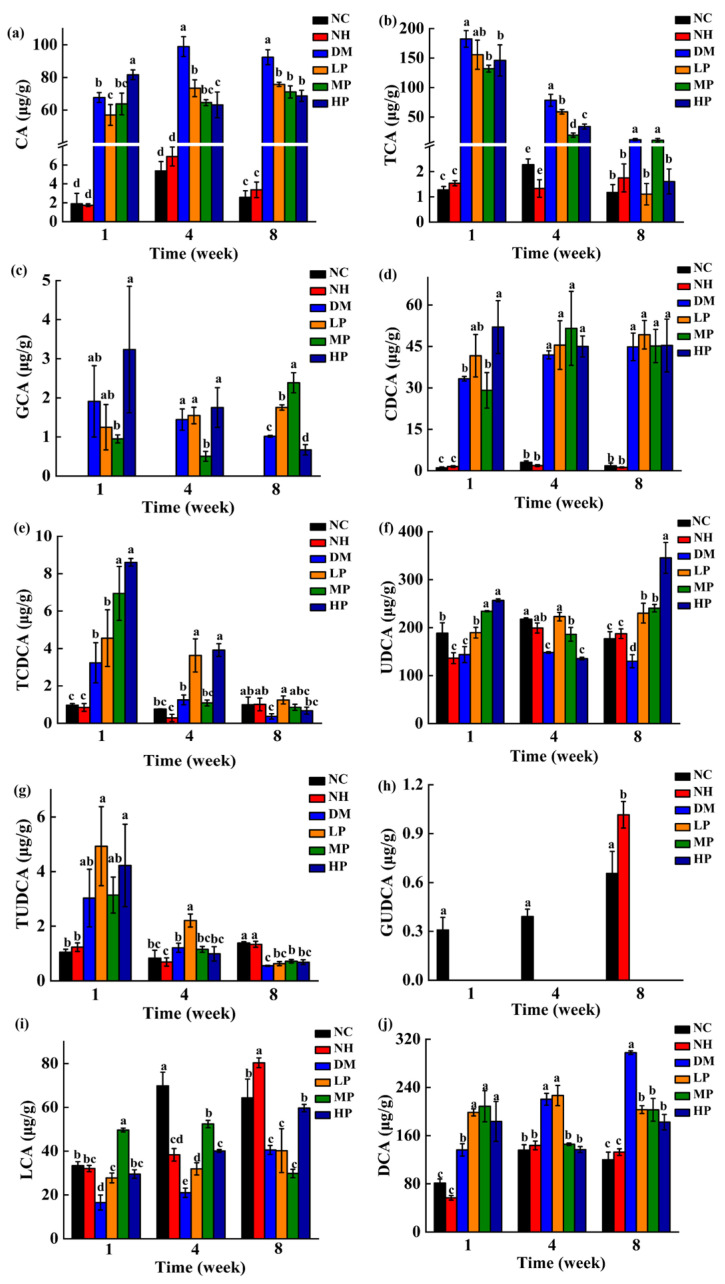
Effects of UPF on the concentrations of bile acid in the feces of rats. (**a**) CA; (**b**) TCA; (**c**) GCA; (**d**) CDCA; (**e**) TCDCA; (**f**) UDCA; (**g**) TUDCA; (**h**) GUDCA; (**i**) LCA; (**j**) DCA. (Note: different lowercase letters indicate significant differences among NC, NH, DM, LP, MP, and HP (one-way ANOVA; LSD or Dunnett’s T3 as appropriate); *p* < 0.05).

Changes in fecal bile acid concentrations across intervention time points (weeks 1, 4, and 8) are presented in Figure 9. Notably, glycocholic acid (GCA) was undetectable in normal rats, while glycoursodeoxycholic acid (GUDCA) was absent in diabetic rats. Consistent with known rodent bile acid metabolism, glycine-conjugated species were present at low concentrations or undetectable in some groups, confirming taurine conjugation as the dominant pathway in rats [35].

No significant differences in bile acid profiles were observed between the NH and NC groups at any time point (*p* > 0.05), indicating that UPF administration did not adversely affect bile acid metabolism in normoglycemic rats. In contrast, the DM group exhibited substantial alterations: concentrations of cholic acid (CA), taurocholic acid (TCA), chenodeoxycholic acid (CDCA), and deoxycholic acid (DCA) were significantly elevated (*p* < 0.05), whereas ursodeoxycholic acid (UDCA) and lithocholic acid (LCA) were significantly reduced (*p* < 0.05) compared to NC. These changes are consistent with adaptations to prolonged high-glucose high-fat diet intake, wherein increased hepatic bile acid synthesis and excretion serve as a compensatory mechanism to mitigate cholesterol accumulation.

UPF intervention significantly attenuated these diabetes-induced alterations. Compared to the DM group, all UPF-treated groups (LP, MP, and HP) showed reduced concentrations of CA, TCA, and DCA (*p* < 0.05), along with increased levels of taurochenodeoxycholic acid (TCDCA), UDCA, tauroursodeoxycholic acid (TUDCA), and LCA (*p* < 0.05). These results suggest that UPF helps restore bile acid homeostasis, potentially reducing the risk of cholesterol supersaturation and supporting metabolic health through targeted modulation of bile acid composition.

### 3.6. Correlation Analysis Between Gut Microbiota and Metabolites

**Figure 10:**
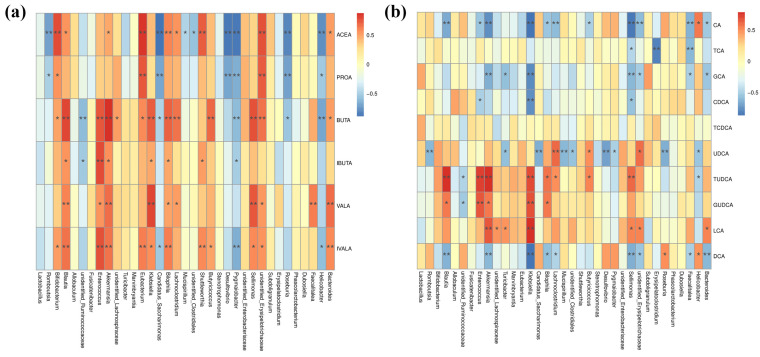
Correlation analysis of the relative abundance of gut microbiota with the concentrations of SCFAs (**a**) and bile acids (**b**). Abbreviations: ACEA, Acetic acid; PROA, Propionic acid; BUTA, Butyric acid; IBUTA, Isobutyric acid; VALA, Valeric acid; IVALA, Isovaleric acid. *, *p* < 0.05; **, *p* < 0.01.

Spearman correlation analysis was performed to evaluate the associations between significantly altered gut microbial genera and the concentrations of SCFAs and bile acids (Figure 10). As illustrated in Figure 10a, multiple significant correlations were identified between specific bacterial genera and SCFAs. The genus *Romboutsia* exhibited significant negative correlations with acetic acid and propionic acid. In contrast, *Bifidobacterium* and *Eubacterium* showed positive correlations with acetic acid, propionic acid, butyric acid, and isovaleric acid. *Blautia* and *Akkermansia* were significantly positively correlated with acetic acid, both forms of butyric acid (butyric and isobutyric acid), and valeric acid (including both valeric and isovaleric acid). *Lachnoclostridium* was positively associated with acetic acid, butyric acid, and valeric acid, while *Butyricicoccus* was significantly correlated with butyric acid and isovaleric acid.

As shown in Figure 10b, the bile acid network was strongly linked to specific taxa. *Romboutsia* exhibited a significant negative correlation with UDCA. *Blautia* was positively correlated with TUDCA/GUDCA and negatively with CA/DCA. *Akkermansia* correlated positively with TUDCA, GUDCA, and LCA, while correlating negatively with CA, GCA, and DCA. *Lachnoclostridium* related positively to UDCA and TUDCA and negatively to CA and DCA. *Butyricicoccus* showed positive links with UDCA and TUDCA and a negative link with CA.

Notably, genera such as *Klebsiella* and *Selimonas*, which are usually linked to pathogenicity or antimicrobial resistance, showed associations with increases in SCFAs and bile acids in this study. This may reflect diabetes-related dysbiosis and the persistence of certain colonizers, which can limit the full effect of UPF on these taxa. Consequently, while UPF improved the overall community, some strongly colonizing genera remained, leading to the observed correlations.

These correlation patterns suggest that UPF may ameliorate diabetic dysbiosis and metabolic dysfunction partly through modulating specific microbe-metabolite interactions, particularly those involving SCFA-producing bacteria and bile acid-transforming microbes, highlighting the potential of dietary intervention in targeting gut microbial metabolism for improved metabolic health.

## 4. Discussion

### 4.1. RS as the Key Bioactive Component in UPF Mediates Anti-Diabetic Effects

RS has emerged as a promising dietary intervention for managing diabetes, with growing evidence suggesting that its beneficial effects are mediated through modulation of gut microbiota and their metabolites [36,37,38]. In the present study, RS was identified as the major functional component of UPF, accounting for approximately 45.2% of its composition (Table S2). We hypothesize that RS serves as the primary bioactive constituent responsible for the anti-diabetic properties observed with UPF intervention. This hypothesis is supported by the fact that RS resists digestion in the small intestine and undergoes microbial fermentation in the colon, yielding biologically active metabolites including SCFAs and secondary bile acids that play crucial roles in metabolic regulation. Our comprehensive analysis demonstrated that UPF intervention significantly improved gut microbiota dysbiosis by enhancing beneficial bacterial populations while suppressing potentially harmful taxa, concurrently modulating multiple metabolite pathways associated with diabetes pathogenesis.

### 4.2. UPF Modulates Gut Microbiota Structure and Potential Microbial Biomarkers

The gut microbiota has gained significant attention as a therapeutic target for metabolic diseases, with particular interest in identifying specific microbial biomarkers associated with diabetes. Our results revealed that UPF intervention significantly reduced the *Firmicutes*/*Bacteroidetes* (F/B) ratio and decreased the relative abundance of *Proteobacteria*. These findings contribute to the ongoing scientific discourse regarding microbial signatures in diabetes. Currently, there is no consensus regarding changes in the F/B ratio in the gut microbiota of patients or animal models with diabetes. Zhao et al. [39] reported a significantly higher F/B ratio in individuals with type 2 diabetes compared with healthy controls. Wang et al. [40] demonstrated that camellia oil alleviated hyperglycemia in db/db mice and reduced the F/B ratio in the treatment group. In addition, Zhang et al. [41] observed that the F/B ratio significantly decreased in type 2 diabetes mellitus mice following intervention with Polysaccharides from *Cynanchum auriculatum* Royle ex Wight. These findings align with our results and suggest that, despite heterogeneity in baseline profiles, a decreased F/B ratio may represent a common response to dietary interventions. Conversely, another study reported a lower F/B ratio in diabetic rats that increased significantly after intervention with moringa (*Moringa oleifera*) *Moringa oleifera Lamarck* seed polysaccharides [42]. This variability may reflect differences in ethnicity, diet, diabetes duration, or methodological approaches. Therefore, this is not a sufficient reason to use the F/B ratio as a precise biomarker of diabetes. Notably, we observed a significant reduction in *Proteobacteria* abundance following UPF intervention, suggesting that *Proteobacteria* may serve as a potential diagnostic and therapeutic target for diabetes management [6]. In summary, we should clearly identify the characteristics of the subject to determine the confounding factors affecting the gut microbiota and explore other diabetes-related microbial bacteria that can be used as biomarkers in future research.

Of particular significance was the specific modulation of individual bacterial genera by UPF intervention. We observed significant increases in multiple beneficial genera including *Lactobacillus*, *Bifidobacterium*, *Blautia*, *Akkermansia*, *Eubacterium*, *Lachnoclostridium*, and *Butyricicoccus*. These genera have well-established roles in maintaining metabolic health: *Lactobacillus* and *Bifidobacterium* are recognized probiotics that improve lipid metabolism and reduce inflammation in diabetic subjects [43,44,45,46]; *Blautia*, *Eubacterium*, and *Butyricicoccus* are important SCFA-producers that enhance intestinal barrier function and glucose homeostasis [47,48,49,50,51,52,53]; *Akkermansia* has emerged as a next-generation beneficial microorganism associated with improved metabolic parameters [54,55]; and *Lachnoclostridium* has demonstrated beneficial effects on insulin sensitivity and lipid metabolism [56].

The enrichment of *Verrucomicrobia* (notably *Akkermansia*) in the DM + UPF-M group (MP) is biologically plausible. *Akkermansia* is a mucin-utilizing bacterium frequently linked to improved gut barrier function, modulation of host metabolic inflammation, and enhanced SCFA profiles [57]. Two mechanisms may explain our observation: (i) substrate availability and dose–response—the medium UPF dose may provide an optimal balance of fermentable substrates and host tolerance that favors *Akkermansia* proliferation, whereas lower doses may be insufficient and higher doses may shift competitive niches; and (ii) bile acid–mucus crosstalk—our bile acid data (e.g., increases in TUDCA and LCA) and SCFA profiles suggest a metabolic milieu that can support *Akkermansia* expansion and mucus turnover [58]. Consistent with prior reports, we observed positive correlations between *Akkermansia* abundance and selected SCFAs in our dataset.

Concurrently, UPF intervention significantly reduced the abundance of *Romboutsia* and *Allobaculum*, genera that have been associated with adverse metabolic profiles. Romboutsia shows positive correlations with triglycerides, cholesterol, and fasting blood glucose [59,60], while Allobaculum abundance increases with high-fat diet consumption and diabetic conditions [61,62]. These findings suggest that RS-rich UPF may ameliorate diabetes through a dual mechanism of promoting beneficial, SCFA-producing bacteria while suppressing diabetes-associated taxa.

### 4.3. UPF Enhances SCFA Production and Restores Metabolic Homeostasis

The fermentation of RS by gut microbiota yields SCFAs that play pivotal roles in metabolic regulation. Our study demonstrated that UPF intervention significantly increased fecal concentrations of acetic, propionic, butyric, isobutyric, and valeric acids in diabetic rats after 8 weeks of intervention. Butyric acid, in particular, deserves special attention due to its crucial role in maintaining colonocyte health, enhancing intestinal barrier function, and regulating glucose metabolism [63]. The significant increase in butyrate levels observed in both normal and diabetic rats suggests that UPF provides a sustainable substrate for butyrogenic bacteria, consistent with previous reports showing that RS intervention increases the fecal butyrate/SCFA ratio [64].

The temporal pattern of SCFA changes is particularly noteworthy. While diabetic rats showed an initial decline in most SCFAs during the first week post-induction, followed by a temporary recovery in weeks 2–3, sustained hyperglycemia led to significant reductions from week 4 onward. UPF intervention progressively reversed this decline, with the most pronounced effects observed in the later intervention period (weeks 4–8). This time-dependent effect underscores the importance of prolonged intervention for achieving meaningful metabolic improvements in chronic conditions like diabetes.

The metabolic benefits of SCFAs are multifaceted: they serve as energy sources for colonocytes; act as signaling molecules through G-protein-coupled receptors; inhibit histone deacetylases; and regulate glucose homeostasis through gluconeogenesis and insulin sensitivity [65]. The increased SCFA production observed in our study, particularly butyrate, likely contributes to the improved metabolic parameters observed in UPF-treated diabetic rats.

### 4.4. UPF Modulates Bile Acid Metabolism and Hydrophilicity

Bile acids represent another important class of microbiota-modulated metabolites that influence glucose and lipid metabolism. Our results demonstrate that UPF intervention significantly altered the bile acid profile, reducing hydrophobic bile acids (CA, DCA) while increasing hydrophilic species (UDCA, TUDCA). This shift toward a more hydrophilic bile acid pool is metabolically favorable, as hydrophobic bile acids are associated with increased intestinal permeability, inflammation, and oxidative stress [66,67], whereas hydrophilic bile acids like UDCA and TUDCA improve insulin sensitivity, reduce inflammation, and protect pancreatic β-cells [68,69].

Additionally, UPF intervention increased the concentration of TCDCA and LCA, suggesting enhanced microbial transformation of primary to secondary bile acids. The increased excretion of secondary bile acids may facilitate cholesterol elimination and contribute to lipid metabolism regulation. These findings align with previous reports that RS alters bile acid metabolism [70,71], though interestingly, our results in diabetic rats differ somewhat from observations in healthy subjects, possibly reflecting the distinct metabolic state of diabetes.

### 4.5. Correlation Between Microbial Shifts and Metabolite Profiles

Spearman correlation analysis revealed significant relationships between specific bacterial genera and metabolic changes. Beneficial bacteria such as *Bifidobacterium*, *Eubacterium*, *Blautia*, *Akkermansia*, *Lachnoclostridium*, and *Butyricicoccus* showed positive correlations with SCFAs and hydrophilic bile acids, while *Romboutsia* correlated negatively with these beneficial metabolites. These correlations suggest that UPF exerts its anti-diabetic effects through structured microbial ecological changes that subsequently modulate metabolic outputs.

The coordinated changes in microbial composition and metabolite profiles support the concept of a microbiota-metabolite axis in diabetes management. Specifically, UPF appears to promote the growth of SCFA-producing bacteria that simultaneously influence bile acid metabolism, creating a synergistic metabolic improvement. This multi-targeted approach may explain the significant metabolic benefits observed with UPF intervention compared to single-target therapies.

## 5. Conclusions

The present study demonstrates that UPF, rich in RS, significantly alleviates diabetes-related metabolic disturbances through targeted modulation of the gut microbiota and its associated metabolites. Our results indicate that UPF effectively ameliorates gut dysbiosis by promoting beneficial bacterial genera—including *Lactobacillus*, *Bifidobacterium*, *Blautia*, *Akkermansia*, *Eubacterium*, *Lachnoclostridium*, and *Butyricicoccus*, while simultaneously enhancing the production of functional metabolites such as SCFAs (acetic acid, propionic acid, butyric acid, isobutyric acid, and valeric acid) and hydrophilic bile acids (UDCA and TUDCA). Spearman correlation analysis further confirmed strong associations between these microbial shifts and metabolic improvements, supporting the presence of a structured microbiota–metabolite axis that mediates the anti-diabetic effects of UPF.

From a functional food perspective, UPF represents a promising dietary strategy for diabetes management by concurrently targeting multiple pathological features: microbial ecology, metabolic output, and systemic glucose and lipid homeostasis. The dose-dependent and time-sensitive effects observed highlight the importance of both sufficient RS intake and sustained intervention to achieve meaningful therapeutic outcomes. These findings not only provide mechanistic insights into how RS-rich foods exert their benefits but also support the use of UPF as a holistic, food-based approach to metabolic health. UPF may be developed along two parallel routes: (1) as a functional ingredient incorporated into everyday foods for glycemic management, and (2) as a concentrated supplement (e.g., pill or sachet) for use in metabolic support in at-risk or diabetic individuals. Further clinical studies are warranted to validate these effects in human populations and to establish practical dietary recommendations for diabetes prevention and management.

## Figures and Tables

**Table 1:** Effects of UPF on α-diversity indexes.

	Shannon	Simpson	Chao1	ACE	Goods_Coverage
NC	5.31 ± 0.26 ^a^	0.92 ± 0.03 ^a^	422.18 ± 29.85 ^a^	423.66 ± 32.29 ^a^	0.9990 ± 0.0000 ^a^
NH	5.51 ± 0.34 ^a^	0.92 ± 0.03 ^a^	415.66 ± 66.96 ^a^	423.23 ± 62.94 ^a^	0.9990 ± 0.0000 ^a^
DM	4.56 ± 0.51 ^b^	0.88 ± 0.03 ^a^	239.27 ± 23.42 ^b^	248.90 ± 23.48 ^b^	0.9990 ± 0.0000 ^a^
LP	4.26 ± 0.25 ^b^	0.89 ± 0.02 ^a^	253.20 ± 49.15 ^ab^	259.77 ± 46.31 ^ab^	0.9994 ± 0.0005 ^a^
MP	4.14 ± 0.50 ^b^	0.87 ± 0.03 ^a^	211.56 ± 9.40 ^b^	212.22 ± 9.49 ^b^	0.9994 ± 0.0005 ^a^
HP	4.36 ± 0.31 ^b^	0.88 ± 0.03 ^a^	225.47 ± 12.74 ^b^	227.12 ± 12.15 ^b^	0.9990 ± 0.0000 ^a^

Note: Different superscript lowercase letters within a column indicate significant differences among groups (one-way ANOVA; LSD or Dunnett’s T3 as appropriate; *p* < 0.05).

## Data Availability

The original contributions presented in the study are included in the article. Further inquiries can be directed to the corresponding authors.

## Supplementary Materials

The following supporting information can be downloaded at: https://www.mdpi.com/article/10.3390/foods14233996/s1, Table S1. Physiological and biochemical indicators of each group at the end of the experiment. Table S2. The nutritional components of unripe plantain flour (in dry basis).

## Abbreviations

The following abbreviations are used in this manuscript:

UPF	Unripe plantain flour
RS	resistant starch
SCFAs	short-chain fatty acids
STZ	streptozotocin
SD	Sprague-Dawley
HSHF	high-sugar high-fat
NC	Normal Control
NH	Normal High-dose
DM	Diabetic Model
LP	Low-dose UPF
MP	Middle-dose UPF
HP	High-dose UPF
PCoA	principal coordinates analysis
LEfSe	Linear discriminant analysis effect size
GCA	glycocholic acid
GUDCA	glycoursodeoxycholic acid
CA	cholic acid
TCA	taurocholic acid
CDCA	chenodeoxycholic acid
DCA	deoxycholic acid
UDCA	ursodeoxycholic acid
LCA	lithocholic acid
TCDCA	taurochenodeoxycholic acid
TUDCA	tauroursodeoxycholic acid
