# Supplementary material for: Dietary Intervention with Resistant Starch-Rich Unripe Plantain Flour Restores Gut Microbiome–Metabolome Axis and Ameliorates Type 2 Diabetes in Rats
Source: Foods. 2025 Nov 21;14(23):3996. doi: 10.3390/foods14233996 (PMC12692605; doi:10.3390/foods14233996)
Supplement: Supplementary file 1 [file foods-14-03996-s001.zip › foods-3928703-supplementary.pdf]

Supplementary Material

Table S1. Physiological and biochemical indicators of each group at the end of the experiment.

	Rate of body weight growth (%)	Average food intakes (g/d/rat)	Average water intake (g/d/rat)	Average 24-h urine volume (g/d/rat)	Serum glucose (mmol/L)	Serum insulin (mU/L)	HOMA-IR
NC	19.66±5.34[a]	22.75±1.73[c]	36.21±1.10[b]	15.64±1.14[b]	8.50±0.45[c]	19.38±1.20[e]	7.16±0.34[e]
NH	15.22±4.94[a]	19.78±0.99[c]	35.13±4.84[b]	15.88±0.21[b]	8.48±0.10[c]	22.04±0.61[e]	8.72±0.89[e]
DM	-14.24±9.60[c]	40.14±0.45[a]	200.64±3.96[a]	183.45±2.63[a]	28.40±2.07[a]	66.15±1.00[a]	72.13±6.29[a]
LP	-5.77±3.38[b]	36.65±2.28[a]	191.95±22.10[a]	167.80±13.57[a]	25.36±0.17[b]	47.27±0.83[b]	61.27±2.93[b]
MP	-11.23±5.16[bc]	39.27±1.14[a]	176.25±17.49[a]	180.81±25.55[a]	25.50±2.22[b]	40.02±1.01[c]	50.81±1.76[c]
HP	-9.32±5.34[bc]	34.27±0.13[b]	184.5±1.38[a]	175.73±8.30[a]	24.44±0.94[b]	33.65±0.79[d]	38.85±3.88[d]

Note: Different superscript lowercase letters within a column indicate significant differences among groups (one-way ANOVA; LSD or Dunnett's T3 as appropriate; $p < 0.05$). HOMA-IR, homeostatic model assessment for insulin resistance index.

Table S2. The nutritional components of unripe plantain flour (in dry basis).

Resistant starch (%)	Total dietary fiber (%)	Carbohydrate (%)	Protein (%)	Fat (%)	Ash (%)	Moisture content (%)
56.09±1.64	5.09±0.76	84.97±3.60	3.51±0.23	0.50±0.00	2.47±0.35	8.55±3.47
